# A meta-analysis of the proportion of animal *Salmonella* isolates resistant to drugs used against human salmonellosis in Ethiopia

**DOI:** 10.1186/s12879-015-0835-x

**Published:** 2015-02-21

**Authors:** Getachew Tadesse

**Affiliations:** Department of Biomedical Sciences, College of Veterinary Medicine and Agriculture, Addis Ababa University, P.O. Box 34, Debre Zeit, Ethiopia

**Keywords:** Animals, Antimicrobial resistance, Ethiopia, Humans, *Salmonella*

## Abstract

**Background:**

The emergence and spread of drug resistant Salmonellae of both human and animal origins are global concerns and worrisome in countries where the risk of infection is high and treatment options are limited. The objective of this study was to estimate the proportions of animal isolates resistant to antimicrobials used against human salmonellosis in Ethiopia.

**Methods:**

Published studies on the antimicrobial resistance features of *Salmonellae* isolated from food animals of Ethiopia were searched in Medline, Google Scholar and the lists of references of articles. Eligible studies were selected by using inclusion and exclusion criteria and data were extracted. The extracted data included the host species, the numbers of isolates and the numbers of ampicillin, co-trimoxazole, chloramphenicol, ceftriaxone and ciprofloxacin resistant isolates. The risks of bias were assessed and the percentages of the variations of the estimates attributable to heterogeneities were quantified. Pooled proportions were estimated by the DerSimonian and Laird random effects model.

**Results:**

Five hundred and fifty four Salmonellae isolated from cattle, camels, sheep, goats and pigs were tested with a variety of antimicrobials. The percentages of the variations attributable to heterogeneities were low for chloramphenicol and ceftriaxone (I^2^ = 0) and high for ampicillin, co-trimoxazole and ciprofloxacin resistance estimates (I^2^ > 75%). The pooled estimate of ampicillin resistant isolates was higher in slaughtered ruminants (17.28%) than in pigs (3.95%), (p < 0.001). The pooled estimates of co-trimoxazole resistant isolates in true ruminants (4.35%) and pigs (1.12%) were not significantly different (p > 0.05). The overall pooled estimates of chloramphenicol and ceftriaxone resistant isolates were 2.24% and 1.25%, respectively. Seven serotypes have been reported to be resistant to antimicrobials uncommonly used in veterinary clinical practice in Ethiopia.

**Conclusions:**

Among Salmonellae of farm animals, there exist strains that are resistant to drugs used in the therapeutic management of human salmonellosis in Ethiopia. Intervention measures should be taken to ensure the prudent use of antimicrobials and curb the spread of high risk strains across the country.

**Electronic supplementary material:**

The online version of this article (doi:10.1186/s12879-015-0835-x) contains supplementary material, which is available to authorized users.

## Background

Non-typhoidal Salmonellae (NTS) are prevalent zoonotic agents and causes of food-borne outbreaks in several countries. The disease is generally considered a self-limiting gastro-enteritis, but antimicrobial therapy is required in severe and invasive infections [1]. However, in present day clinical practice drug resistant strains have posed a problem because infections are often associated with increased morbidity and mortality [2]. The problem is worrisome in developing countries where the risk of infection is high and treatment options are limited.

Of particular concern is the occurrence of strains resistant to drugs of critical importance to human health such as the extended-spectrum cephalosporins and the fluoroquinolones [3-5]. There have been several reports from Africa and Asia of NTS of animal origin resistant to first and second line drugs used for the therapeutic management of human salmonellosis [6-10]. *Salmonella* ser. Kentucky ST198-X1, originally identified in the Mediterranean area in 2009, has become a global threat as it showed resistance to several antimicrobials including the extended spectrum cephalosporins, carbapenems and azithromycin [3].

Of the NTS serotypes in Ethiopia, *Salmonella* ser. Concord is the most common serotype of the human isolates reported to be resistant to third generation cephalosporins [11], and *S*. Kentucky is the most frequently reported ciprofloxacin resistant serotype of animal origin [12,13]. Since the prevalence of *Salmonella* in domestic food animals of Ethiopia ranges from 7.07% (95% CI = 2.05, 16.17) in cattle to 43.81% (95% CI = 38.89, 48.85) in pigs [14], the risk to humans appears high because of the low living standard of the population, the closeness between animals and humans, and the habits of consumption of raw animal products in a significant segment of the population [15]. Furthermore, the management of the disease is complicated due to the limited diagnostic facilities and therapeutic alternatives in most clinical settings of the country [16]. Despite the importance of the disease, surveillance and monitoring systems are not in place and the pharmaco-epidemiology of the bacteria is not described. A quantitative synthesis of previous studies’ estimates could, however, provide an insight into the magnitude of the problem and the comparative importance of food animals as potential sources of high risk strains. Such information could be of significant importance in clinical practice and development of intervention measures aimed at reducing the risk associated with the disease. The objectives of this study were to estimate the proportions of *Salmonella* isolates of animal origin that are resistant to drugs used in the management of human salmonellosis in Ethiopia by using meta-analytical methods. The outcomes of interest were the proportions of ampicillin, co-trimoxazole, chloramphenicol, ceftriaxone and ciprofloxacin resistant isolates.

## Methods

### Eligibility criteria

To be eligible a study (i) had to be published; (ii) had to be written in English; (iii) had to be cross sectional and at least two types of samples were examined to detect *Salmonella* from each sampled animal; (iv) had to describe the microbial isolation, identification and antimicrobial sensitivity test methods; and (v) had to report the number of tested isolates and the number of isolates resistant or sensitive to one or more of the following drugs: ampicillin, co-trimoxazole, chloramphenicol, ceftriaxone and ciprofloxacin.

### Search and selection of studies

**Figure 1:**
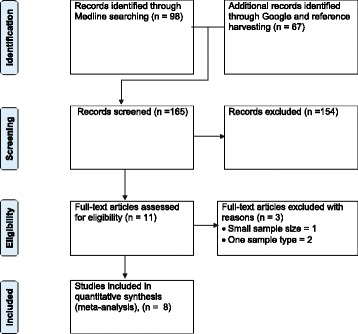
**A flow diagram of the selection of eligible studies.**

Figure 1 shows the search and selection of eligible studies. The search strategy is described in a previous study [11]. Briefly, published studies were searched in Medline, Google scholar and the lists of references of articles. The last search was done on December 10, 2014. A total of 165 studies were found, and 154 were excluded because the titles and abstracts were not relevant to the outcomes of interest. Of the 11 articles screened for eligibility, one study was excluded because the number of tested isolates was two, and two studies were excluded because the isolates were from one sample type. Eight studies were eligible for quantitative syntheses [12,13,17-22].

### Data extraction

From each eligible study, the first author, year of publication, year of study, location of study, host species, study design, number of animals, sampling method, types of samples, number of samples, microbial isolation/identification and antimicrobial test methods and protocols, number of isolates, and numbers of ampicillin, co-trimoxazole, chloramphenicol, ceftriaxone, ciprofloxacin and multi-drug resistant isolates and serotypes were extracted. Multi-drug resistance was defined as resistance to three or more drugs. The study level proportions were derived from the extracted data. The data was extracted by TG.

### Data analysis

To produce conservative estimates, a zero reported for the numbers of isolates either resistant or susceptible to a drug or drugs was imputed as 0.5 [23]. The study level proportion (p) and standard error (se) were calculated by the following formulae: p = r/n and se = √ p(1-p*)*/n: where r = number of resistant isolates and n = number of tested isolates. To normalize the distribution of the data, the study level estimates were logit transformed [24,25]: lp = ln[p/(1 − p)], where lp = logit event estimate; ln = natural logarithm and p = study level estimate. The variance of the logit event estimate was calculated by using the following formula: v(lp) = 1/(np) + 1/[n (1 − p)], where v = variance and n = sample size.

### Assessment of bias

The within study bias was assessed on the basis of the qualities of the sampling design, the microbial isolation and identification methods, and the antimicrobial test methods. A study was considered to be of a good quality if the sample size was determined by considering expected prevalence and desired precision of the estimate; animals were sampled by using a probability sampling method; bacterial isolation and identification were done by using steps that involved pre enrichment, selective enrichment and biochemical tests. The across studies bias (small study effects) was assessed by funnel plots [26], and the Duval and Tweedie non-parametric ‘trim and fill’ linear random model was used to calculate unbiased estimates [27].

### Heterogeneity analyses

The Galbraith plot [28], Cochran’s Q test and inverse variance index (I^2^) [29] were used to assess heterogeneity of estimates. Because of the low power of the Q test to detect heterogeneity among small number of studies, a significant heterogeneity was accepted if the ratio of the Cochran’s Q and the degree of freedom (Q/df) was less than one. The inverse variance index was used to quantify the percentage of the variation attributable to heterogeneity, and I^2^ values of 25%, 50% and 75% were considered as indicators of low, moderate and high heterogeneity respectively. A subgroup analysis was done if the heterogeneity was moderate to substantially high (I^2^ > 50%), and on the basis of feel of relative homogeneity as true ruminant, pseudo-ruminant and non-ruminant data.

### Quantitative synthesis

Pooled logit event estimates were calculated by the DerSimonian and Laird random effects model [30], and back-transformed to proportions: p = e^lp^/(e^lp^ + 1), where e = the base of the natural logarithm. Influence analyses were done to assess the robustness of pooled estimates. A study was considered to be highly influential if the pooled estimate without it was outside the 95% confidence bounds of the overall mean. The Yates corrected Chi Square and Fisher exact two tailed test were used to assess the significance of differences in proportions [31,32] and the strength of association was measured by odds ratio (OR). Alpha was set at 0.05.

Microsoft Office Excel 2007 was used to calculate study level proportions, logit event estimates, standard errors and to back-transform logit event estimates to proportions. Epi Info™ (Version 3.5.1, Center for Disease Control, CDC, USA) was used to compare groups. All other analyses were done by using Stata (Version 11.1, Stata Corp, College Station, Texas).

## Results and discussion

The study was conducted according to the guideline on Preferred Reporting Items for Systematic Reviews and Meta-Analyses (PRISMA) [33]. The PRISMA Checklist was used to ensure inclusion of relevant information (see Additional file 1). Most, if not all published reports on the subject were considered in the study. However, despite the importance of NTS of animal origin at global and national levels, and the large livestock population of the country, the number of studies that addressed the occurrence and distribution of drug resistant Salmonellae was small.

### Characteristics of the eligible studies

Table 1 presents the characteristics of the eligible studies. The studies were carried out between 1999 and 2010 in Central, Northern, and Eastern Ethiopia on slaughtered cattle, sheep, goats, camels and pigs, and dairy cattle. The exact origins of the study populations were not described in but one study [22]. A total of 6486 samples taken from 1506 animals were examined and the sensitivities of 554 isolates to a variety of antimicrobials were tested. Most slaughtered ruminants are raised in the extensive production system, and the study animals were collected from various markets of the lowland, midland and highland animal production areas of the country. Pigs and dairy cattle were from small scale intensive/semi-intensive farms in central Ethiopia. Therefore, regardless of the potential location and genetic differences, it is reasonable to assume that the study populations represent the farm animal populations of the country.

**Table 1:** **Characteristics of the eligible studies**

						**Number positive (%)**				
**Author**	**Year**	**Location**	**Host**	**Ni**	**Amp**	**Sxt**	**Chl**	**Cro**	**Cip**	**MDR**
[17]	1999-2000	DZ	Ct	25	13 (52)	1 (4)	0 (0)	0 (0)	0 (0)	13 (52)
[18]^a^	2001-2002	DJ	Cm	116	28 (24.14)	24 (20.69)	4 (3.45)	2 (1.72)	0 (0)	33 (28.45)
[19]	2003-2004	AM	Sg	22	4 (18.18)	2 (9.09)	1 (4.55)	0 (0)	0 (0)	4 (18.18)
[12]	2004-2005	DZ	Pg	94	2 (2.13)	1 (1.06)	0 (0)	0 (0)	30 (31.91)	66 (70.21)
[13]^b^	2004-2005	AA	Pg	173	8 (4.62)	2 (1.16)	2 (1.16)	0 (0)	7 (4.05)	55 (31.79)
[20]^c^	2005-2006	DZ	Ct	75	0 (0)	0 (0)	0 (0)	0 (0)	0 (0)	3 (4)
[21]	2006-2007	BD	Ct	28	2 (7.14)	Nt	1 (3.57)	Nt	Nt	3 (10.71)
[22]	2010	AA	Dc	21	21 (100)	0 (0)	2 (9.52)	0 (0)	0 (0)	14 (66.67)

AA = Addis Ababa; AM = Addis Ababa and Modjo; Amp = ampicillin; BD = Bahir Dar; Chl = chloramphenicol; Cip = ciprofloxacin; Cm = slaughtered camels; Cro = ceftriaxone; Ct = slaughtered cattle; Dc = dairy cattle; DJ = Diredawa and JiJiga; DZ = Debrezeit; MDR = multi-drug resistant; Ni = number of tested isolates; Nt = not tested; Pg = slaughtered pigs; Sg = slaughtered sheep and goats; Sxt = co-trimoxazole.

^a^Data on ceftiofur was substituted for ceftriaxone.

^b^The numbers of co-trimoxazole, chloramphenicol and ciprofloxacin resistant isolates was extracted from the narrative part of the results and discussion section.

^c^Isolates from holding pens were excluded.

### Risks of within study bias

Table 2 presents the sampling, bacterial isolation and antimicrobial test methods. Sample sizes were determined by considering expected prevalence and desired precision of the estimate in three studies [13,20,22] but not reported in others. Sampling of animals was random in five studies [12,19-22], and all animals presented for slaughter in the sampling days were sampled in three studies [13,17,18]. From each sampled animal, two or more types of samples were examined. The sample matrices included gastro-intestinal tract (GIT) contents (rumen, small intestine, caecum and feces), liver, tongue, spleen, hide swabs, abdominal and diaphragmatic muscles, carcass swabs and hand swabs at flaying and evisceration. Bacterial isolation was done according to the guidelines of the International Organization for Standardization (ISO) with some modifications, and steps that included pre-enrichment, selective enrichment and biochemical tests were employed in all studies. The analytical unit considered in seven studies was 25 grams (<25 grams in a few cases where samples were inadequate), and one gram of feces and one milliliter of milk in one study [22]. The sensitivities of isolates to panels of 8 to 24 antimicrobials (aminoglycosides, cephalosporins, co-trimoxazole, nitrofurans, penicillins, phenicols, polymixins, quinolones, sulphonamides, teracyclines, trimethoprim and co-trimoxazole) were tested by using the dilution [12,13,17-20] and diffusion methods [21,22]. Antimicrobial sensitivity test results were interpreted according to the interpretative standards of the National Committee for Clinical Laboratory Standards (NCCLS) [12,13,17-19,21,22] and the Clinical Laboratory Standards Institute (CLISI) [20]. Accordingly, the within study bias was considered unimportant for the following reasons. First, the study animals were derived from various agro-climatic zones and farms but not from a specific farm or location. Second, as animals were sampled in small batches and across several months, the sampling guarantees a wider coverage of the target population of slaughtered ruminants and pigs. Third, the microbial isolation methods employed in all studies were similar. The smaller analytical units from dairy cattle samples [22] might have underestimated the recovery of the bacteria but not the relative proportions of drug resistant or susceptible isolates. Moreover, despite antimicrobial susceptibility data generated based on dilution and diffusion tests are qualitatively comparable [34], the breakpoint levels of susceptibility and resistance to ciprofloxacin differ among the studies. The higher breakpoint level of susceptibility to ciprofloxacin (>0.125 μg/ml) compared to the performance standards (0.06 μg/ml) of the 24^th^ CLISI guideline (M100-S24) suggests underestimation of the prevalence of ciprofloxacin resistant isolates. On the other hand, the determination of resistance based on a susceptibility break point level alone [12,13] irrespective of the resistant breakpoint level might have led the classification of isolates that displayed an intermediate resistance phenotype in the resistant category. Similarly, one dairy cattle isolate that reportedly displayed intermediate resistance to ciprofloxacin [22] might have been resistant, and other isolates reportedly susceptible might have been intermediately resistant or resistant. In general, with the exception of the evolving interpretative standards for ciprofloxacin resistance, the similarities of the methods are great enough to justify pooling and obtain unified conclusions on the proportions of mono-drug and multi-drug resistant isolates.

**Table 2:** **Sampling, isolation and antimicrobial test methods**

**Author**	**Sampling method**	**Number of animals**	**Sample matrices**	**Number of samples**	**Method of isolation** ^**b**^	**Number of drugs**	**Drug test** ^**c**^
[17]^a^	all	323	f, m, ad	1292	ISO	17	mic
[18]^a^	all	119	f, m, ad, l, s	714	ISO	17	mic
[19]	rs	204	f, m, ad, l, s	1224	ISO	24	mic
[12]	rs	101	c, m, ad, l, t	501	ISO	24	mic
[13]^a^	all	278	c, m, cs	833	ISO	24	mic
[20]	rs	100	r, c, m, cs, h, hs	788	ISO	24	mic
[21]	rs	186	i, m, l, cs	744	ISO	8	dzi
[22]	rs	195	f, ml	390	ISO	10	dzi

ad = abdominal and diaphragmatic muscles; cs = carcass swab; rs = random sampling; c = caecal contents; dzi = diameter of zone of inhibition; f = feces; h = hide; hs = hand swabs at flaying and evisceration; i = intestinal contents; l = liver; m = mesenteric lymph nodes; mic = minimum inhibitory concentration; ml = milk; s = spleen; r = rumen contents; t = tongue.

^a^All animals slaughtered on each sampling day were sampled.

^b^The bacterial isolation and identification methods were according the International Organization for Standardization (ISO-6579, 1998–2002) [12,13,17-22] and Quinn *et al*. (Clinical Veterinary Microbiology, printed from1994-2004) [12,13,17-21], and GSS (Global *Salmonella* Surveillance) and NHS (National Health Service for Wales) [22].

^c^The interpretative standards were according to the National Committee for Clinical Laboratory Standards (NCCLS, 1990–2005) [12,13,17-19,21,22] and Clinical Laboratory Standards Institute (CLSI, 2005) [20]; the susceptibility break point levels for ciprofloxacin resistance were < = 0.125 μg/ml [12,13]; < = 0.5 μg/ml [18] and < = 1 μg/ml [17] but not reported in three studies[19,20,22], and the resistance break point levels were > = 1 μg/ml [18] and > = 4 μg/ml [17] but not tested [12,13] and not reported in others [19,20,22].

### Risks of across studies bias

**Figure 2:**
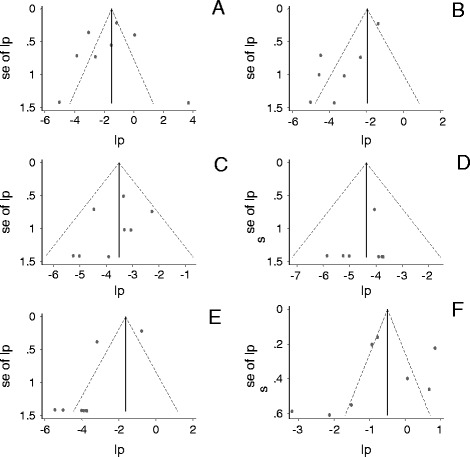
**Funnel plots of the logit event estimates (lp) of ampicillin (A), co-trimoxazole (B), chloramphenicol (C), ceftriaxone (D), ciprofloxacin (E) and multi-drug (F) resistant isolates.**

Figure 2 presents funnel plots of the logit event estimates. Asymmetries and outliers were detected in estimates of ampicillin, co-trimoxazole, ciprofloxacin and multi-drug resistant isolates, but unbiased pooled proportions were not estimated by the Duval and Tweedie method. Across study biases (small study effects) were ruled out for three reasons. First, the numbers of animals (> = 100) and the numbers of samples (> = 390) were fairly large. Second, the studies appear to have been published due to their appropriateness and the importance of drug resistant *Salmonella* at a global level rather than due to large estimates. Third, the funnel plots’ skewing patterns suggest true heterogeneity of the study populations.

### Heterogeneity of estimates

**Figure 3:**
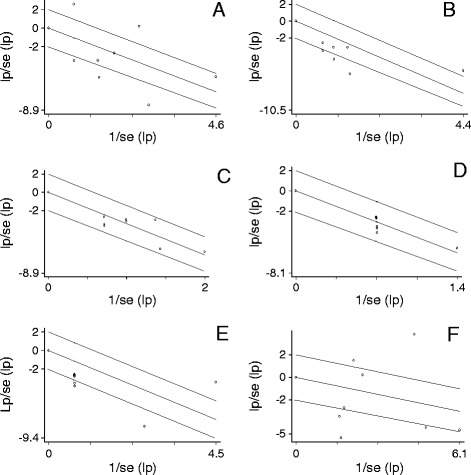
**Galbraith plots of the logit event estimates (lp) of ampicillin (A), co-trimoxazole (B), chloramphenicol (C), ceftriaxone (D), ciprofloxacin (E) and multi-drug (F) resistant isolates.**

The Galbraith plots displayed heterogeneities of the proportions of ampicillin, co-trimoxazole, ciprofloxacin and multi-drug resistant estimates (Figure 3). The percentages of the variations attributable to heterogeneities were substantially high (I^2^ > 75%) for ampicillin, co-trimoxazole, ciprofloxacin and multi-drug resistant isolates, but low (I^2^ < 25%) for chloramphenicol and ceftriaxone resistant isolates. Omitting camel [18] and pig isolates [12,13] that highly contributed to the heterogeneities of co-trimoxazole and ciprofloxacin resistance estimates, respectively, yielded low I^2^ values (I^2^ < 25%). However, omitting dairy cattle isolates [22] that highly contributed to the heterogeneity of ampicillin resistance estimates did not substantially shrink the inverse variance index (I^2^ > 75%). In a further subgroup analyses, the I^2^ values of the proportions of ampicillin resistant isolates in slaughtered ruminants and ciprofloxacin resistant isolates in pigs were substantially high (I^2^ > 75%). The heterogeneity analyses of the estimates of ampicillin, co-trimoxazole, ciprofloxacin and multi-drug resistant isolates indicate substantial variations within and/or between host species. These variations could have been due to differences in the magnitude of exposure of the hosts to a drug or drugs or a higher occurrence of specific drug resistant serovars regardless of exposure to drugs. In contrast, the lower between-study variations in the proportions of chloramphenicol and ceftriaxone resistant isolates (<25%) reflect the similarities of the occurrence of such isolates across different host species.

### Forest plots

**Figure 4:**
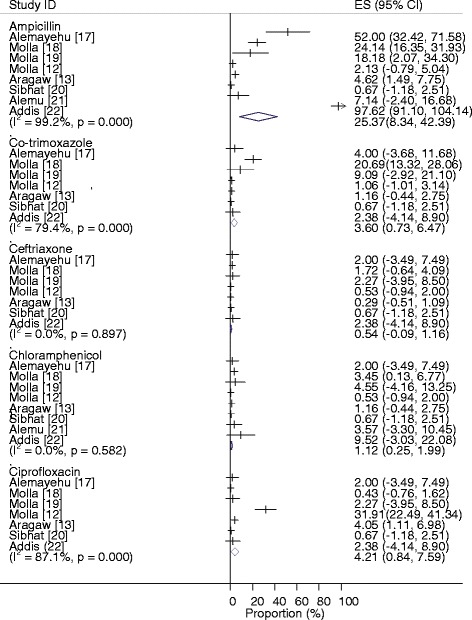
**Forest plots of the proportions of drug resistant isolates.**

Forest plots of the proportions of *Salmonella* resistant to each of the drugs are presented in Figure 4. The highest proportions of ampicillin and co-trimoxazole resistant isolates were recorded in dairy cattle and camels, respectively, and the lowest in slaughtered cattle. The lowest and highest proportions of chloramphenicol resistant isolates were recorded in pigs and dairy cattle, respectively. Ceftiofur and ciprofloxacin resistant Salmonellae were isolated from camels and pigs, respectively.

### Pooled proportions

The pooled estimates are presented in Table 3. The overall occurrence of isolates resistant to antimicrobial groups known to have been used in the management of animal diseases was generally higher than the occurrence of isolates resistant to drugs uncommonly used in veterinary clinical practice in Ethiopia. The pooled estimate of ampicillin resistant isolates was higher in slaughtered ruminants [17.28% (95% CI = 7.28, 37.36)] than in pigs [3.95% (95% CI =2,14, 7.20)], [X^2^ = 22.85; p = 0.000; OR = 4.87 (95% CI = 2.37, 10.23)]. The pooled estimates of co-trimoxazole resistant isolates in true ruminants [4.35% (95% CI = 1.57, 11.45)] and pigs [1.12% (95% CI = 0.36, 3.43)] were not significantly different (p > 0.05). The overall pooled estimates of chloramphenicol and ceftriaxone resistant isolates were 2.24% (95% CI = 1.38, 3.6) and 1.25% (95% CI = 0.52, 2.98), respectively, and that of ciprofloxacin resistant isolates in pigs was 12.55% (95% CI = 1.34, 60.32). All pooled estimates lie within the 95% confidence bounds of the respective overall means.

**Table 3:** **Pooled proportions and heterogeneity estimates of drug resistant isolates**

**Host**	**Drug**	**Pooled estimate (%)**		**Heterogeneity**		
		**p (95% CI)**	***Z-p***	**Q** ***-p***	**Q/df**	**I** ^**2**^
Ruminants	Amp^a^	17.28 (7.28, 37.36)	0.003	0.000	5.21	80.8
	Sxt^b^	4.35 (1.57, 11.45)	0.000	0.370	1.5	4.5
	MDR^a^	19.12 (8.40, 37.87)	0.003	0.000	6.3	84.1
Pigs	Amp	3.95 (2,14, 7.20)	0.000	0.317	1.00	0.2
	Sxt	1.12 (0.36, 3.43)	0.000	0.946	0.0	0.0
	Cip	12.55 (1.34, 60.32)	0.107	0.000	29.32	96.6
	MDR	50.98 (17.52, 83.59)	0.961	0.000	33.9	97.0
All animals	Chl	2.24 (1.38, 3.61)	0.000	0.366	1.09	8.3
	Cro	1.25 (0.52, 2.98)	0.000	0.883	0.39	0.0

Amp = ampicillin; Chl = chloramphenicol; Cip = ciprofloxacin; Cro = ceftriaxone; df = degrees of freedom; I^2^ = inverse variance index; Q = Cochran’s Q; Q*-p* = probability of Cochran’s Q test; Sxt = co-trimoxazole; Z-*p* = probability of Z test.

^a^The estimate is for slaughtered ruminants.

^b^The estimate is for true ruminants.

Despite ampicillin being in the list of drugs for use in Ethiopia [35], it was not one of the top three commonly prescribed antimicrobials in public and private veterinary clinics in Addis Ababa [36], and its availability and use in animals is generally similar throughout the country. Therefore, the occurrence of ampicillin resistant isolates in all host species could partly be a cross-resistance phenomenon with penicillin G- a drug frequently prescribed alone or in combination with streptomycin. However, as bovine mastitis is a major health problem in the dairy sector of Ethiopia [37-40], clinical cases are often treated with antimicrobials in the form of injectables, or as intra-mammary suspensions that may contain ampicillin or other β-lactams. Therefore, the absolute resistance of dairy cattle isolates to ampicillin (100%) could by and large be a result of direct resistance to the drug and cross resistance to other β-lactams. Similarly, the higher occurrence of co-trimoxazole resistant isolates in camels (20.69%) could have been due to the frequent use of the sulfonamides for the treatment of camel salmonellosis in the pastoral areas [41]. In general, even though some isolates might have been of human origin and others emerged elsewhere and introduced into the country, the imprudent use of antimicrobials in animals could be implicated as one of the factors that promoted the occurrence drug resistant Salmonellae of animal origin in the country. Studies have shown a slack drug use policy, non-adherence to rational antimicrobial use guideline, and several stakeholders that include regulatory bodies, drug importers, professionals, para-professionals as well as livestock owners might have contributed to the misuse of drugs and occurrence of resistant pathogens. For instance, in one study, the veterinary drugs’ import subsector was characterized by lack of basic monitoring and data collection systems, poor quality drugs, unethical practice, a thriving unofficial trade over the borders and unlicensed importations [42]. Additionally, informal drug sellers have mushroomed, and drugs and substandard agents are commonly sold in open air markets and ordinary shops [43]. Furthermore, the diagnosis of diseases is generally presumptive; a few antimicrobials (penicillin G, penicillin-streptomycin combination and oxytetracycline) are frequently prescribed with little justifications [36], and across the Ethiopia-Kenya border, community based workers account for 83.8% (140/167) of the health care service providers [44]. Moreover, animal traders use antimicrobials as blanket treatments at times when their animals display signs of ill health, and oxytetracycline residues were detected in 71.3% (274/384) each of beef and kidney samples of cattle slaughtered in three abattoirs in Central Ethiopia [45].

Resistance to drugs uncommonly used in the veterinary clinical practice of Ethiopia (chloramphenicol, third generation cephalosporins and ciprofloxacin) was a feature of seven serotypes (Table 4). The proportion of these isolates was lower than the proportion of isolates resistant to antimicrobial classes commonly used for the management of diseases in animals, and with the exception of *S*. Typhimurium strains, all these serotypes were resistant to only one of these drugs.

**Table 4:** **Serotypes resistant to drugs uncommonly used in clinical practice**

**Serotype**	**Host**	**No. tested**	**Chl**	**Cro**	**Cip**	**Authors**
*S.* Kentucky	Pg	23	0	0	20	[12,13]
*S.* Blockley	Pg	8	0	0	8	[12]
*S.* Enteritidis	Pg, Sg	17	0	0	5	[12,13,17,19]
*S.* Typhimurium^a^	Pg, Cm, Ct, Sg	36	7	1	2	[13,17-21]
*Salmonella* ser*.* Infantis^b^	Pg, Cm, Ct	12	1	0	0	[13,18,21]
*S.* Anatum	Pg, Cm, Ct	62	0	1	0	[12,13,18,20]
*S.* I:9,12:-:-	Pg	2	0	0	2	[12]
Total		160	8	2	37	

Chl = chloramphenicol; Cip = ciprofloxacin; Cro = ceftriaxone; Cm = slaughtered camels; Ct = slaughtered cattle; Pg = slaughtered pigs; Sg = Slaughtered sheep and goats.

^a^The number of isolates tested with third generation cephalosporins was 31 [13,17-20].

^b^The number of isolates tested with third generation cephalosporins was 7 [13,18].

All chloramphenicol resistant isolates were MDR of which six were resistant to the β-lactams, aminoglycosides and sulphonamides (Table 5). Resistance to chloramphenicol was shown by *S*. Typhimurium strains, and in a study on the drug resistance features of chicken isolates in Ethiopia, whilst 88.9% (24/27) of the *S.* Typhimurium isolates were resistant to chloramphenicol, the rest six serotypes (53/53) were susceptible [46]. *S*. Typhimurium is the third most frequently isolated serotype from human clinical samples in Ethiopia [11], and it is a common cause of invasive disease [47,48] with high mortality in AIDS patients in Africa [49].

**Table 5:** **MDR serotypes resistant to phenicols, cephalosporins and fluoroquinolones**

**Drug**	**Serotype**	**MDR pattern**	**Host**	**Nri**	**Author**
Chl	*S.* Typhimurium^a^	ChlStrTet	Cattle	1	[21]
		AmpChlSmxSptStrTet	Camel	1	[18]
		AmpAmc ChlFenSmxSptStrTet	Camel	2	[18]
		AmpAmcCefChlSptStrSulSxtTmp	Shoat	1	[19]
		AmpAmcChlCipFenNalSptStrSulTet	Pig	2	[13]
	*S.* Infantis	ChlColSpeTet	Camel	1	[18]
Cro	*S.* Anatum	CftSptStr	Camel	1	[18]
	*S.* Typhimurium	AmpAmc CftSmxSptStrTet	Camel	1	[18]
Cip	*S.* Kentucky	AmpAmcCipNal	Pig	1	[13]
		CipNalSptSulTet	Pig	2	[12]
		CipNalSptStrSulTet	Pig	10	[12]
		CipGenNalSptStrSulTet	Pig	2	[12]
		AmpAmcCipNalStrSulTet	Pig	1	[13]
		AmpAmcCefCipNalStrSulTet	Pig	1	[13]
		AmpAmcCipGenNalSptStrSulTet	Pig	2	[13]
	*S.* Blockley	CipKanNalNeoNitStrTet	Pig	7	[12]
		AmpCipKanNalNeoNitStrTet	Pig	1	[12]
	*S.* Enteritidis	CipNalNit	Pig	5	[12]
	*S.* I:9,12:-:-	CipNalNit	Pig	2	[12]
	*S.* Typhimurium^a^	AmpAmcChlCipFenNalSptStrSulTet	Pig	2	[13]

Amp = ampicillin; Amc = amoxicillin-clavulanic acid; Cef = Cephalothin; Chl = chloramphenicol; Cft = ceftiofur; Cip = ciprofloxacin; Col = colistin; Cro = ceftriaxone; Fen = florphenicol; Gen = gentamicin; Kan = kanamycin; Nal = nalidixic acid; Neo = neomycin; Nit = nitrofurantoin; Nri = number of resistant isolates; Smx = sulfamethoxazole; Spt = spectinomycin; Str = streptomycin; Sul = sulfisoxazole; Sxt = co-trimoxazole; Tet = tetracycline; Tmp = trimethoprim.

^a^The same pig isolates were resistant to chloramphenicol and ciprofloxacin.

Four of the chloramphenicol resistant isolates were *S.* Typhimurium DT104 isolated from camels and pigs. *S.* Typhimurium DT104 was isolated from a human sample in Ethiopia [50]; it is an internationally important pathogen [51], and regardless of the sources, isolates with an ACSSuT (ampicillin, chloramphenicol, streptomycin, sulfonamide and trimethoprim) phenotype have the same gene cassettes [52], and genetic differences at different times [53], locations [54] and hosts [55] have been reported to be little.

Despite the fact that the third generation cephalosporins have not been used for the management of animal diseases in Ethiopia, two camel isolates, one each of *S*. Typhimurium and *Salmonella* ser. Anatum, were resistant to ceftiofur (Table 4). Although *S*. Concord is a predominant human isolate [11] and highly resistant to the extended spectrum cephalosporins in Ethiopia [4,5,56], it was not isolated neither from camels nor from other food animal species. Therefore, the clonal spread of NTS serotypes [51] and the extensive trans-boundary movements of camels as beasts of burden suggest that these isolates might have been introduced into Ethiopia from neighboring countries.

Resistance to ciprofloxacin was a feature of five serotypes of which *S*. Kentucky accounted for 54.05% (20/37) of these isolates (Table 4). Ciprofloxacin resistant *S*. Kentucky strains have been isolated from chicken [57], a French traveler who visited Ethiopia [3] and from chicken meat and minced beef collected from markets [58] but not demonstrated in other studies on slaughtered ruminants [17-21], chicken samples [46,59], minced beef and mutton [60], and humans [56,61] in Ethiopia. The isolation of a few *S*. Kentucky from market samples alone [57,58] suggests either cross contamination with pigs or contaminated pork or a limited occurrence of the serovar in other host species. In contrast, in the USA [62] and in Europe [63], *S*. Kentucky was one of the top three serovars most frequently isolated from broiler carcasses.

Ciprofloxacin resistant *S*. Kentucky reportedly originated in Egypt and spread to several African countries, the Middle East and Europe [3,57] and might have been introduced in to Ethiopia with imported animals as there have been no screening tests at the farm of origin or after importation. The other ciprofloxacin resistant strains (*Salmonella* ser*.* Blockley, *Salmonella* ser*.* Enteritidis, *S.* Typhimurium and *Salmonella* ser.I:9,12:-:-) were all isolated from pigs and might have emerged elsewhere and introduced with imported animals. Although pig production has been limited to Central Ethiopia for a long time, there have been recent efforts [64,65] to increase pork production which may facilitate the rapid spread of these high risk strains across the country.

The selection pressure created by the commonly used antimicrobials could be one of the factors that promoted the occurrence of MDR strains of ruminant origin in proportions as high as 66.7% in dairy cattle. Similarly, the proportion of MDR *Salmonella* isolates of human origin in Ethiopia was estimated at 79.6% and the odds in the 2000s was 19 times higher than before the 1990s [66]. As drug resistant genes may occur in integrons, the use of antimicrobials could offer a selective advantage to strains that possess several resistant gene cassettes. In one study on the genotypic features of 98 Ethiopian MDR Salmonellae, Class I integrons were identified in 53.1% of the isolates of which 61.5% had integron-associated gene cassettes that predominantly confer resistance to aminoglycosides and trimethoprim [50]. On the whole, notwithstanding the limited genotypic evidence, the considerable occurrence of MDR isolates (19.12% and 50.98% in slaughtered ruminants and pigs respectively and 66.7% in dairy cattle) coupled with the absence of a strict drug use policy suggests the potential for an increase in the proportion of drug resistant Salmonellae.

Despite sparse data on the pattern and frequency of application of antimicrobials in ruminants, the prevalence levels of oxytetracycline residues in slaughtered cattle (71.3%) [45], as well as the occurrence of bovine mastitis [37-40] and common use of antimicrobials to treat clinical cases are suggestive of an association between antimicrobial use and the prevalence of MDR isolates in ruminants. In Europe, the occurrence of antimicrobial resistant *Streptococcus pneumoniae* (indicator) was positively correlated with the use of *β*-lactams and macrolides [67]. Although the emergence and propagation of drug resistant isolates is a complex issue, it is reasonable to take for granted that the higher proportion of MDR isolates in Ethiopian pigs than in ruminants could be due to a higher occurrence of certain serovars rather than a more frequent exposure of pigs to antimicrobials. For instance, *Salmonella* ser. Hadar and *S*. Kentucky were the most frequent pig isolates [14], and 98.04% (100/102) of the MDR strains of these serotypes were tetracycline resistant. Therefore, as swine production in Ethiopia started with exotic animals, most MDR pig isolates might have been introduced with imported animals.

The MDR profiles of the serotypes resistant to chloramphenicol, ceftiofur and ciprofloxacin are presented in Table 5. These isolates represent 25% (44/176) of the MDR strains of which 75% (33/44) were resistant to more than five antimicrobials and 97.7% (43/44) were susceptible to co-trimoxazole. Resistance to potentiated amoxicillin was exhibited by chloramphenicol (5/8), ceftiofur (1/2) and ciprofloxacin (5/35) resistant isolates. The ciprofloxacin resistant isolates were resistant to nalidixic acid, and all but two isolates were susceptible to third generation cephalosporins.

Table 6 presents MDR serotypes susceptible to phenicols, third generation cephalosporins and fluoroquinolones. These isolates accounted for 75% (132/176) of the MDR strains of which *Salmonella* ser. Braenderup (24/24) was resistant to six or more drugs. Resistance to ampicillin was displayed by 31.1% (41/132) of these isolates and all were resistant to sulfonamides but not to potentiated amoxicillin.

**Table 6:** **MDR serotypes susceptible to phenicols, third generation cephalospoins and fluoroquinolones**

**Serotype**	**MDR pattern**	**Host**	**Nri**	**Author**
*S.* Braenderup	AmpSptStrSulSxtTmp	Pig	1	[13]
	AmpSmxSpeStrSxtTmp	Camel	19	[18]
	AmpSpeStrSxtTetTmp	Camel	1	[18]
	AmpColSmxSpeStrSxtTmp	Camel	3	[18]
*Salmonella* ser*.* Haifa^a^	AmpStrTet	Cattle	2	[21]
*S.* Hadar	NitStrTet	Pig	80	[12,13]
*Salmonella* ser*.* Heidelberg	StrSmxTet	Camel	1	[18]
	StrSmxSpeTetTmp	Camel	1	[18]
*Salmonella* ser*.* Kiambu	AmpStrSulSxtTetTmp	Pig	1	[12]
*Salmonella* ser*.* Mishmarhaemek	AmpSmxTic	Cattle	11	[17]
	AmpCefSmxTic	Cattle	1	[17]
*Salmonella* ser*.* Newport	StrSulTet	Cattle	2	[20]
*Salmonella* ser*.* Reading	StrSulTet	Sheep/goat	2	[19]
*Salmonella* ser*.* Saintpaul	ColSpeStrTmp	Camel	1	[18]
*Salmonella* I:6,7:eh:-	AmpColSmxSpeStrSxtTmp	Camel	1	[18]
*S.* Typhimurium	StrSulSxtTetTmp	Sheep/ goat	1	[19]
	AmpSmxTicSxt	Cattle	1	[17]
Salmonella 1:6.8:–:enx	NitStrTet	Pig	2	[13]
Salmonella1:6.8:z10:	NitStrTet	Pig	1	[13]

Amp = ampicillin; Cef = cephalothin; Col = colistin; Nit = nitrofurantoin; Nri = number of resistant isolates; Smx = sulfamethoxazole; Spt = spectinomycin; Str = streptomycin; Sul = sulfisoxazole; Sxt = co-trimoxazole; Tet = tetracycline; Tic = ticarcillin; Tmp = trimethoprim.

^a^The sensitivities of the isolates to potentiated amoxicillin were not tested.

### Implications of the study

Backyard slaughtering is a widespread practice in the country; raw beef and goat meat are considered delicacies for most urban inhabitants and milk is often consumed raw in the rural community. The raw meat consumption rate peaks in festivities and individuals may contract *Salmonella* during such occasions*.* Therefore, as drug sensitivity tests are not carried out and therapy is empirical in many clinical settings of the country, chloramphenicol, co-trimoxazole, ceftriaxone and ciprofloxacin retain apparent utility against strains of true ruminant origin.

The analysis demonstrates the overall drug resistance problem and the need for an effective policy and intervention measures. First, as there are strains resistant to drugs of critical importance to human health, inclusion of other drugs in the essential drug list is important to manage severe and life threatening infections. Second, the prohibition of selling breeding pigs from herds infected with high risk strains could reduce the rate of establishment of the strains across farms and regions. In addition, as *Salmonella* can survive for about ten months in the environment [68], proper management of pig waste could lessen the spread and transmission of high risk strains to other animals and humans. Third, importing semen and use of artificial insemination could serve the purpose of daunting introduction of high risk strains, and if live animals are imported, restriction to specific locations and ascertaining the absence of high risk strains prior to their release as breeders could deter their spread. Fourth, as antimicrobial misuse could result in the emergence and propagation of resistant pathogens [69,70], intervention measures to ensure the prudent use of drugs and curb further escalation of the problem are needed. Educational programs to health care providers could improve adherence to rational drug use [71,72]. Therefore, updating animal health care service providers, improving diagnostic facilities and measures against illegal access to antimicrobials could help reduce the impact of antimicrobial use. Fifth, public education on risks associated with the consumption of raw animal products, and hygiene could decrease the risks of transmission of *Salmonella* and other food-borne pathogens to humans. Moreover, as the numbers of intensive farms in the country is small, initiating a system of surveillance and monitoring of high risk strains is important to devise alternative control and preventive strategies.

The number of studies that have addressed drug resistant NTS of animal origin in Ethiopia is small and the genotypic features including the temporal and spatial distributions of the serotypes are not adequately described. Further studies are needed to describe the pharmaco-epidemiology of *Salmonella* of animal origin as such data could help to establish a nationwide pharmaco-vigilance system and contain drug resistant high risk strains at their origin.

### Limitations of the study

The small number of studies was the major limitation in the meta-analysis. Pooled proportions were not calculated for ampicillin resistant isolates of dairy cattle and co-trimoxazole resistant isolates of camels. In addition, despite substantial heterogeneities in the proportions of ampicillin resistant isolates of slaughtered ruminants and ciprofloxacin resistant isolates of pigs, sub-group analyses were not done because the numbers of studies were reduced to one in further sub-groupings. In addition, because of dearth of data, the potential effects of breed, exact origin and management of animals as risk factors were not assessed, and seeing that all studies but one were conducted before 2007, the prevalence of drug resistant isolates might have changed. Moreover, because of the need to refine susceptibility testing reports, the breakpoint levels of ciprofloxacin susceptibility and resistance have been reevaluated and changed, and a new susceptible-dose- dependent category has been included in the recent version of the CLISI guideline (M100-S24). Therefore, the pooled estimate of the proportion of ciprofloxacin resistant *Salmonella* does not reflect the likely prevalence of ciprofloxacin resistant isolates in the country.

## Conclusions

All species of food animals are human exposure sources of drug resistant *Salmonellae,* and there exist strains that are resistant to first and second line drugs used in the therapeutic management of human salmonellosis. Austere intervention measures should be taken to ensure the prudent use of antimicrobials and curb the spread of high risk strains, and further studies are needed to describe the pharmaco-epidemiology of NTS at the national level in Ethiopia.

## Additional file

Additional file 1:
**PRISMA Checklist.**
